# Factors associated with late diagnosis of breast cancer among women in Botswana

**DOI:** 10.4102/phcfm.v17i1.4829

**Published:** 2025-11-18

**Authors:** Punishment P. Chibatamoto, Chester Kalinda, Moses J. Chimbari

**Affiliations:** 1 Discipline of Public Health, School of Medicine, College of Health Sciences, University of KwaZulu-Natal, Durban, South Africa; 2 Bill and Joyce Cummings Institute of Global Health, University of Global Health Equity (UGHE), Kigali, Rwanda

**Keywords:** Botswana, women, breast cancer, stage of diagnosis, patient factors

## Abstract

**Background:**

Breast cancer is a public health issue in Botswana. Associations of patient-level factors with late breast cancer diagnosis are not well understood. This may explain why there are many cases of late diagnosis.

**Aim:**

We assessed patient-level factors associated with late breast cancer diagnosis among women in Botswana.

**Setting:**

The study was conducted at four designated cancer public health facilities in Botswana.

**Methods:**

A cross-sectional hospital-based survey questionnaire was administered to 211 adult women (15 September 2023 – 15 December 2023). Descriptive statistics, Chi-square/Fisher’s exact test and logistic regression were performed using StataNow 18 SE to analyse the association of patient factors with late diagnosis for breast cancer.

**Results:**

Forty-six per cent (*n* = 90) of women studied presented with advanced cancer at the first stage of diagnosis. Occupation (*χ*^2^ = 9.0342; *p* = 0.029) and age at first full-term pregnancy (*χ*^2^ = 6.3287; *p* = 0.042) were associated with late diagnosis at bivariate analysis. With univariate analysis, being single (odds ratio [OR]: 0.184, 95% confidence interval [CI]: 0.036–0.932) and formally employed (OR 3.395, 95% CI: 1.467–7.860) were associated with late diagnosis. Multivariate analysis identified second-degree family history as a major predictor of late cancer diagnosis among women (adjusted odds ratio [AOR]: 0.340, 95% CI: 0.129–0.893).

**Conclusion:**

Almost half (45.91%, *n* = 90) of the study participants presented with advanced stages of breast cancer at the time of initial diagnosis. While we did not study all women in Botswana, the geographical spread of our sample reflects a countrywide problem. We recommend scaling-up cancer awareness campaigns for improved benefits of early breast cancer screening and diagnosis.

**Contribution:**

We identified patient level factors associated with late breast cancer diagnosis among women studied in Botswana. Thus, our study informs an awareness campaign for reducing cases of breast cancer late diagnosis.

## Introduction

Breast cancer is a major public health problem globally,^1^ with an estimated 2.3 million new cases reported in 2022.^2^ Furthermore, breast cancer was reported as the fifth leading cause of mortality among cancers, accounting for 685 000 women.^3^ The occurrence of breast cancer among women has been attributed to complex interactions among genetic, hormonal, population structure, lifestyle and environmental factors.^4^ Emerging evidence suggests that mortality because of breast cancer in most parts of sub-Saharan Africa is partly because of late diagnosis,^5^ as 80% of African women are reported to have been diagnosed with advanced cancer stages (III or IV) at first diagnosis.^6^

Breast cancer stages are classified using the American Joint Committee on Cancer (AJCC) TNM system.^7^ The TNM system determines the size and location of the primary *tumour* (T), the involvement of the lymph *nodes* (N) and the extent of *metastasis* (M). The non-invasive ductal carcinoma *in situ* (DCIS) phase is classified as stage 0, and stages I through IV are used for invasive breast cancer. The greater the number is, the more the cancer has spread. Early diagnosis is associated with stages 0, I and II, and advanced stages (III and IV) are associated with late diagnosis.^7^ Late diagnosis of breast cancer leads to a reduced survival rate.^8^

Breast cancer is increasingly becoming a public health burden in Botswana.^9^ According to the World Health Organization (WHO), of all reported cancers (2317) reported in 2022, female breast cancer accounted for 11.1% (*n* = 257) new cases, making it the second most common cancer after cervical cancer.^2^ Furthermore, the country’s age-adjusted breast cancer incidence rate increased from 17.5 per 100 000 women in 2018 to 20.0 per 100 000 women in 2020,^10^ whereas the age-adjusted mortality rate of breast cancer increased from 8.64 per 100 000 women to 20.0 per 100 000 women in 2020,^11^ suggesting the need for improved optimal disease-management strategies.

Several studies have shown that early diagnosis is critical for improving the survival rates and quality of life of women with breast cancer.^12,13^ However, some studies have indicated that between 72% and 90% of women present with advanced stages of breast cancer at first diagnosis in Botswana.^14,15^ The high prevalence of late diagnosis is a cause for concern for a country with health expenditures equivalent to 6.1% of the gross domestic product^16^ and free delivery of health services to all citizens through a hierarchical network of public health facilities. There is a need for comprehensive approaches to gain a thorough understanding of the factors associated with the late diagnosis of breast cancer in Botswana to develop evidence-based policies and strategies for improved screening and early detection programmes. We therefore sought to determine the patient-level factors associated with late breast cancer diagnosis among women at four designated oncology centres in Botswana.

## Research methods and design

### Study design

A cross-sectional hospital-based survey utilising a descriptive-analytical approach was used to identify patient-level factors associated with late breast cancer diagnosis among adult women in Botswana.

### Setting

The study was conducted at all four cancer-designated public health facilities across the country. The study participants included women with clinically confirmed diagnosis of breast cancer seeking breast cancer care at the four public health facilities. The four health facilities are located in the southern (Gaborone city), northern (Francistown city), central (Serowe town) and northwestern (Maun town) part of the country. The four designated cancer public health facilities coordinate multidisciplinary care and oncology-related services and provide linkages with human immunodeficiency virus (HIV) care and follow-up care for all cancer patients in the country. The treatment of women diagnosed with cancer at either public hospitals or private hospitals is covered by the government healthcare system.

### Study population and sampling strategy

The study population consisted of all outpatient women diagnosed with breast cancer at all four designated public health cancer centres. The sample size was calculated via the following formula:


$$n=\left(\left[t^{2}pq]/[d^{2}\right]\right)$$ [Eqn 1]

Where *n* = minimum sample size needed; *t* = 1.96 value of confidence level desired; *p* = 16.1% national female breast cancer prevalence^17^; *q* = 1-*p*; and *d* = 5% precision (desired degree of accuracy).

Based on these, the calculated sample size was 207. Assuming a non-response rate of 10%, the final sample size was 227 participants. The sample size for each of the four cancer designated centres was proportionally determined according to the number of women receiving breast cancer care at each site. The proportions were based on historical hospital breast cancer data. On average, each of the public referral hospital and district hospital provides breast health services to 5–10 women per week.

### Inclusion criteria

Any outpatient adult woman aged (18 years and above), not very ill or mentally incapacitated but with clinically confirmed breast cancer and a tumour stage documented in the patient medical records stored at any of the four designated public health cancer centres in Botswana was included in the study.

### Data collection

A structured face-to-face interviewing questionnaire was used to collect data from sociodemographic data (residential place, religion, marital status, highest level of education, occupation, age and height), inherited risk or genetic predisposition factors or hormonal factors (family history, age at menarche, age at first childbirth parity, duration of breastfeeding and age at menopause) and personal behaviours or choices or lifestyle patient factors (smoking, alcohol, breastfeeding history, parity and use of modern contraceptives). The questionnaire was pre-tested on 20 women with breast cancer at facilities which did not participate in the study. The questionnaire validation outcomes were face validity, content validity, reliability and internal consistency. The pre-test demonstrated that the questionnaire accurately and consistently captured the intended patient factors related to breast cancer.

Additionally, clinical data of the patients interviewed were extracted from their hospital records at the four participating public health facilities. The clinical data included anthropometric measurements and tumour stage of the patient at the time of initial diagnosis. All questionnaires were anonymous and labelled according to participant numbers. A total of 211 adult black female citizens of Botswana with confirmed breast cancer were interviewed between 15 September 2023 and 15 December 2023. The interviews were conducted weekly by four well-trained nurses at each of the four public oncology clinics.

### Data analysis

Descriptive statistics were used to summarise the sociodemographic factors and present the clinical characteristics of the study participants. The diagnoses were categorised as early (stages I and II) or late (stages III and IV) in accordance with the AJCCs TNM classification system.^7^ This was later converted into a binary variable, with early diagnosis being coded as ‘0’ and late diagnosis being coded as ‘1’. The associations between social-demographic and clinical characteristics and the stage of the cancer were determined via the Chi-square test or Fisher’s exact test. Sociodemographic and clinical characteristics that are associated with the stage of the cancer were further explored via multivariable logistic analysis to determine their influence on the stage of the cancer, as in the case of a similar analysis.^18^ Furthermore, variables with a *p*-value < 0.2 in the univariate analysis were included in the multivariate analysis.^19^ The model goodness of fit was tested via the Hosmer–Lemeshow test and link test, as demonstrated elsewhere.^20^ All analyses were performed via StataNow 18 SE.

### Ethical considerations

Ethical approval was obtained from the Health Research Development Committee (HRDC) of the Ministry of Health (MOH), Botswana on 08 June 2020 with ethical application reference number HPDME 13/18/1, as well as University of KwaZulu-Natal (UKZN) Biomedical Research Ethics Committee (BREC), South Africa on 16 September 2020, ethical application reference number BREC/00001596/2020. The research participants were informed that the study would not expose them to any harm (physical, psychological, social or economic) and that there would not be any anticipated benefits that may result from the research. They were also informed that their participation would help create knowledge and innovations for improved management of breast cancer. Informed consent was obtained and women who did not give their consent for participation were excluded from the study. Subsequent to the information provided, written consent was obtained from all participants prior to their enrolment in the study.

## Results

### Sociodemographic characteristics of the study participants

**FIGURE 1:**
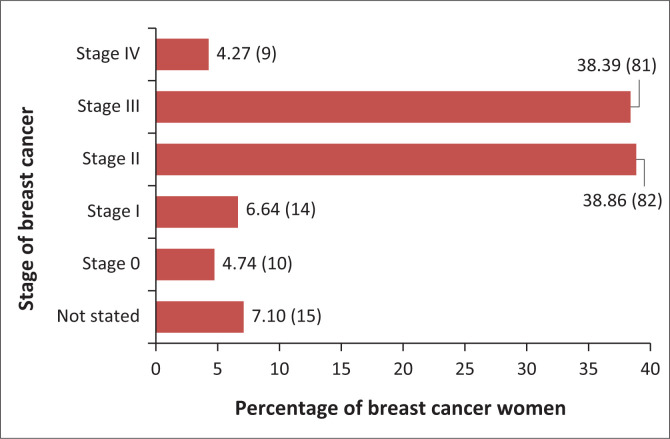
Stage at diagnosis of women with breast cancer who participated in the study, Botswana 2024 (*N* = 211).

Among the 227 study participants enrolled, 92.3% (*n* = 211) participated in the study. The ages of the 211 participants ranged from 20 years to 95 years, and their median age was 52 years (interquartile range [IQR]: 43–64). The majority (52.0%; *n* = 110) of the 211 participants were single. In addition, 28.6% (*n* = 60) had never attended school, whereas 55.9% (*n* = 118) were unemployed. The medical records of 15 (7.1%) of the 211 women with clinically confirmed breast cancer did not indicate the stage at first diagnosis, as shown in Figure 1.

Furthermore, of the 211 women with clinically confirmed breast cancer, 4.7% (*n* = 10) presented with a non-invasive stage at first diagnosis, 6.6% (*n* = 14) presented with stage I at first diagnosis and 38.9% (*n* = 82) presented with stage II at first diagnosis. In addition, of the 196 women with a known breast cancer stage at first diagnosis, 90 (45.91%) presented late at the time of first diagnosis.

### Factors associated with the late stage of diagnosis

Among the demographic and clinical characteristics studied, occupation (χ^2^ = 9.0342; *p* = 0.029) and age at first full-term pregnancy at the time of diagnosis (χ^2^ = 6.328; *p* = 0.042) were associated with the stage of breast cancer at first diagnosis (Table 1).

**TABLE 1:** Associations of patient-level factors with late breast cancer diagnosis among women (bivariate analysis), Botswana, 2024.

Patient level factor	Early stage		Late stage		Stage of diagnosis (*p*-value)
	*n*	%	*n*	%	
**Sociodemographic factors**					
**Age at first diagnosis (years)**	-	-	-	-	0.995
20–38	16	55.17	13	44.83	-
39–57	52	54.74	43	45.26	-
58–76	33	53.23	29	46.77	-
77–95	5	55.56	4	44.44	-
**Marital status**	-	-	-	-	0.078
Cohabiting	2	22.22	7	77.78	-
Widowed	16	44.44	20	55.56	-
Married	26	53.06	23	46.94	-
Single (never married)	62	60.78	40	39.22	-
**Highest level of education**	-	-	-	-	0.448
None	29	51.79	27	48.21	-
Primary	32	49.23	33	50.77	-
Secondary	26	65.00	14	35.00	-
Tertiary	19	54.29	16	45.71	-
**Occupation**	-	-	-	-	0.029*
Unemployed	62	59.62	42	40.38	-
Formal employment	10	30.30	23	69.70	-
Informal employment	8	61.54	5	38.46	-
House wife	19	52.78	17	47.22	-
**Personal behaviours and lifestyle factors**					
**Alcohol history**	-	-	-	-	0.249
No	75	51.72	70	48.28	-
Yes	30	61.22	19	38.78	-
**Smoking history**	-	-	-	-	0.765
No	86	54.09	73	45.91	-
Passive smoker	7	46.67	8	53.33	-
Yes	13	59.09	9	40.91	-
**Average duration of breast feeding per child (months)**	-	-	-	-	0.307
0–24	66	50.77	64	49.23	-
25–48	6	50.00	6	50.00	-
No stated	34	62.96	20	37.04	-
**History of breast feeding**	-	-	-	-	0.172
No	22	64.71	12	35.29	-
Yes	84	51.85	78	48.15	-
**Number of biological children**	-	-	-	-	0.424
None	19	57.58	14	42.42	-
1–4	67	56.30	52	43.70	-
5–9	20	45.45	24	54.55	-
**Family planning method**	-	-	-	-	0.717
No	85	53.46	74	46.54	-
Yes	21	56.76	16	45.92	-
**Age at first full-term pregnancy (years)**	-	-	-	-	0.042 *
14–19	25	56.82	19	43.18	-
20–36	18	38.30	29	61.70	-
Not sure	63	60.00	42	40.00	-
**Inherited factors**					
**First degree family history**	-	-	-	-	0.624
Unknown	95	53.37	83	46.63	-
Known	11	61.11	7	38.89	-
**Duration of menstrual cycle**	-	-	-	-	0.699
Not sure	96	54.55	80	45.45	-
3–7 days	10	50.00	10	50.00	-
**Second degree family history**	-	-	-	-	0.051
Unknown	87	51.18	83	48.82	-
Known	18	72.00	7	28.00	-
**Personal history of breast cancer**	-	-	-	-	0.572
Not known	8	66.67	4	33.33	-
One breast	94	53.41	82	46.59	-
Other cancers	1	100.00	0	0.00	-
Two breasts	3	42.86	4	57.14	-
**Size of bra**	-	-	-	-	0.091
Don’t know	73	50.34	72	49.66	-
30–38	24	60.00	16	40.00	-
40–46	9	81.82	2	18.18	-
**Menarche age (years)**	-	-	-	-	0.090
Not sure	79	58.09	57	41.91	-
12–22 years	27	45.00	33	55.00	-
**Regular or irregular menstrual cycle**	-	-	-	-	0.094
Irregular	10	66.67	5	33.33	-
Regular	30	65.22	16	34.78	-
Not stated	66	48.89	69	51.11	-
**Type of chronic disease**	-	-	-	-	0.573
None	42	57.53	31	42.47	-
Asthma	1	33.33	2	66.67	-
Hypertensive	30	53.57	26	46.43	-
Diabetes	7	70.00	3	30.00	-
HIV	25	47.17	28	52.83	-

HIV, human immunodeficiency virus.

* , Significance *p* < 0.05.

As shown in Table 2, late breast cancer diagnosis was associated with being single (odds ratio [OR]: 0.184, 95% confidence interval [CI]: 0.036–0.932) and formally employed (OR: 3.395, 95% CI: 1.467–7.860). Further analysis via a multivariate model suggested that a known second-degree family history was a significant predictor of a late cancer diagnosis, with 76% of those with a known history (adjusted odds ratio [AOR]: 0.340, 95% CI: 0.129–0.893) being less likely to have a late diagnosis than those with an unknown second-degree family history of cancer.

**TABLE 2:** Patient-level factors associated with late diagnosis of breast cancer among women in univariate and multivariate analyses, Botswana, 2024.

Patient level factors at first time diagnosis	Univariate analysis			Multivariate analysis		
	Odds ratio (OR)	*p*-value	95% confidence interval (CI)	Adjusted odds ratio (AOR)	*p*-value	95% confidence interval (CI)
**Marital status**						
Cohabiting (Ref)	-	-	-	-	-	-
Widowed	0.357	0.236	0.065–1.962	-	-	-
Married	0.252	0.106	0.048–1.341	-	-	-
Single (never married)	**0.184**	0.041 *	**0.036–0.932**	-	-	-
**Occupation**						
Unemployed (ref)	-	-	-	-	-	-
Formal employment	**3.395**	0.004*	**1.467–7.860**	-	-	-
Informal employment	0.923	0.894	0.282–3015	-	-	-
House wife	1.321	0.475	0.616–2.832	-	-	-
**Second degree family history**						
Unknown (ref)	-	-	-	-	-	-
Known	0.408	0.057	0.162–1.026	**0.340**	0.028*	**0.129–0.893**
**Bra cup size**						
Not stated (ref)	-	-	-	-	-	-
30–38	0.676	0.281	0.332–1.377	0.767	0.498	0.356–1.651
40–46	0.225	0.062	0.047–1.079	-	-	-
**Menarche age (years)**						
Not sure (ref)	-	-	-	-	-	-
12–22	1.693	0.091	0.918–3.124	-	-	-
**Menses**						
Irregular (ref)	-	-	-	-	-	-
Regular	1.067	0.918	0.311–3.661	-	-	-
Not stated	2.091	0.199	0.679–6.442	-	-	-
**Age at first full-term pregnancy (years)**						
14–19 (ref)	-	-	-	-	-	-
20–36	2.120	0.079	0.917–4.899	-	-	-
Not sure	0.877	0.719	0.430–1.789	-	-	-

* , Significance *p* < 0.05.

## Discussion

The stage at first diagnosis is a critical measure for improved cancer care and increases the likelihood of survival of female breast cancer patients. Our study revealed that almost half (45.91%) of women presented with advanced stages (III/IV) of breast cancer at the time of first diagnosis, which is consistent with findings from Egypt, with a 47.5% late diagnosis rate^21^; Togo, with 55.1%^22,23^ and Sudan, with 50.1%.^24^ Although the prevalence of late diagnosis in Botswana may be lower than that reported within regions such as Zimbabwe (> 80%) as reported in 2023^25^; Tanzania at 80% in 2022^26^; Kenya at 80% in 2022^27^; Zambia at 66% in 2020^28^ and South Africa at 63.3% in 2022,^9^ late diagnosis still remains a challenge necessitating increased health education and cancer screening campaigns across the country. Overall, our study suggests that routine screening efforts, awareness campaigns and health education need to be encouraged at all levels of healthcare.

The findings from our study also suggest that progress has been made over the years in enhancing cancer care among women in Botswana. Earlier studies have suggested that the prevalence of late diagnosis is variable but greater than that reported in this study. For example, a study conducted in 2013 suggested that 90% of women presented with late-stage breast cancer.^15^ This percentage decreased to 56.6% in 2018^29^ but increased to 64.7% in 2022.^9^

Although this finding reflects some significant strides in early diagnosis efforts, Botswana’s rates are still considered high compared with rates of late-stage diagnosis of breast cancer in high-income countries. Our study revealed that late breast cancer diagnosis was associated with being single and formally employed. Furthermore, this study shows that second-degree breast cancer family history is protective against late cancer diagnosis. Knowledge of family cancer history is critical for early diagnosis, as demonstrated in other studies.^30,31,32^

Studies have shown that women with a family history of breast cancer are more likely to develop bilateral cancer at a younger age and these cases also tend to have a less favorable prognosis.^33,34^ Thus, service providers can utilise ‘documented family cancer history tools’ to identify women at increased risk of cancer and help develop personalised plans for cancer prevention and early detection, as demonstrated elsewhere.^35^ This documented family cancer history tool can be used to identify people at increased risk of cancer and help personalise plans for cancer prevention and early detection. The utilisation of family cancer histories calls for strengthened communication among individuals, families and healthcare providers, as demonstrated in other studies.^36,37^

Although this study indicated that second-degree breast cancer family history is associated with late breast cancer diagnosis, earlier studies reported that other sociodemographic, personal behaviours or choices and inherited or genetic predisposition factors are associated with stage at the first time of breast cancer diagnosis and survival.^38^ It is important to prioritise early identification of all patient-level risk factors to enable proactive clinical management. Awareness of these patient-level factors can facilitate early diagnosis of breast cancer and ultimately prevent exposure to these modifiable factors. Public health education helps women understand the importance of early detection and treatment.

Reducing the incidence of late-stage cancer has become critical in enhancing the incidence of breast cancer among several African governments. For example, the Africa Centers for Disease Control and Prevention (Africa CDC) is supporting African Union member states in comprehensively strengthening systems with a strong focus on multisectoral prevention of breast cancer and other non-communicable diseases (NCDs). The African governments are guided by a framework with six African CDC strategic priorities.^39^ Furthermore, governments are following the recommended pathway with a focus on: (1) patients’ awareness and access to care; (2) clinical evaluation, diagnosis and staging and (3) access to subsequent treatment.^40^

In Botswana, the government has taken deliberate and strategic action to move the health promotion and disease prevention aspects of NCDs to the National AIDS Council (NACA), now called the National AIDS and Health Promotion Agency (NAHPA). This move elevated the importance of NCDs by allowing them to cross-cut high-level priority status in the Office of the President. This also provides an opportunity for NAPHA to leverage lessons learned during the HIV and AIDS epidemic response. These include governance issues, financing, human resources, products and technologies, information systems and community engagement, which will be translated into the fight against NCDs.

Recently, Botswana has taken several initiatives in addressing gaps in prevention and support services. These gaps include shortages of specific trained staff, long delays in cancer detection and diagnosis and shortages of cancer medications and weak monitoring and evaluation systems.^41^ The initiatives implemented to address the gaps include the establishment of a national, comprehensive cancer care and prevention programme through the following:

- development of a national strategy with a focus on the four major NCDs, which account for 82% of NCD-related deaths (cancers, cardiovascular diseases, diabetes, chronic respiratory disease), as well as the four common modifiable risk factors they are associated with (smoking, harmful use of alcohol, unhealthy diet and physical inactivity)^17^
- development of standardised national cancer control and treatment guidelines for most prevalent cancers in Botswana^42^
- conducted a comprehensive cancer care and prevention needs assessment at the four public oncology centres in the public health system to understand their strengths and weakness^43^
- development of a course on clinical management (cancer basics, cancer screenings, treatment modalities for common cancers, palliative care, patient navigation and referral pathways within Botswana health systems)
- establishment of a National Cancer Pathology Working Group established in 2020 to improve pathology and laboratory medicine services for cancer throughout Botswana’s health system
- establishment of a global oncology fellowship programme that provides fellows with global oncology experience for the improvement of cancer care and prevention initiatives in Botswana.

This study underscores the importance of continued advocacy and education in improving the health-seeking behaviour of women in the context of breast cancer. This study suggests the creation of awareness and enhanced screening strategies as critical strategies for early breast cancer diagnosis. Addressing the challenge of late breast cancer diagnosis in Botswana requires a multifaceted approach through awareness creation and enhanced screening. Health initiatives and policies should focus on emphasising the importance of early detection and screening for breast cancer among women. In addition, breast cancer screening programmes should be implemented across all geographic regions of Botswana, with a particular focus on areas that target underserved populations. These initiatives could lead to earlier identification of breast cancer cases and better patient outcomes.

## Conclusion

Our findings indicate that almost half (45.91%) of the study participants presented with advanced stages of breast cancer at the time of initial diagnosis, indicating a need to improve awareness, knowledge and benefits of breast cancer screening and diagnosis. While we did not study all women in Botswana the geographical spread of our sample reflects a countrywide problem. Hence, we recommend that Botswana upscales cancer awareness campaigns.
